# Investigating Individual Pre-trauma Susceptibility to a PTSD-Like Phenotype in Animals

**DOI:** 10.3389/fnsys.2019.00085

**Published:** 2020-01-14

**Authors:** Khadijah S. Alexander, Rebecca Nalloor, Kristopher M. Bunting, Almira Vazdarjanova

**Affiliations:** ^1^VA Research Service, Charlie Norwood VA Medical Center, Augusta, GA, United States; ^2^Department of Pharmacology and Toxicology, Augusta University, Augusta, GA, United States

**Keywords:** PTSD, RISP model, susceptibility, rats, hippocampus, medial prefrontal cortex, immediate early genes, risk factors

## Abstract

Post-Traumatic Stress Disorder (PTSD) is a complex condition that develops after experiencing a severe emotional trauma, with or without physical trauma. There is no known cure and evidence-based treatments, which are effective in reducing symptoms, have low retention rates. It is therefore important, in addition to seeking new therapeutics, to identify ways to reduce the likelihood of developing PTSD. The fact that some, but not all, individuals exposed to the same traumatic event develop PTSD suggests that there is individual susceptibility. Investigating susceptibility and underlying factors will be better guided if there is a coherent framework for such investigations. In this review, we propose that susceptibility is a dynamic state that is comprised of susceptibility factors (before trauma) and sequalae factors (during or after trauma, but before PTSD diagnosis). We define key features of susceptibility and sequalae factors as: (1) they are detectable before trauma (susceptibility factors) or during/shortly after trauma (sequalae factors), (2) they can be manipulated, and (3) manipulation of these factors alters the likelihood of developing PTSD, thus affecting resilience. In this review we stress the importance of investigating susceptibility to PTSD with appropriate animal models, because prospective human studies are expensive and manipulation of susceptibility and sequalae factors for study purposes may not always be feasible. This review also provides a brief overview of a subset of animal models that study PTSD-related behaviors and related alterations in endocrine and brain systems that focus on individual differences, peri- and post-trauma. Attention is drawn to the RISP model (Revealing Individual Susceptibility to a PTSD-like Phenotype) which assesses susceptibility before trauma. Using the RISP model and expression of plasticity-associated immediate early genes, *Arc* and *Homer1a*, we have identified impaired hippocampal function as a potential susceptibility factor. We further discuss other putative susceptibility factors and approaches to mitigate them. We assert that this knowledge will guide successful strategies for interventions before, during or shortly after trauma that can decrease the probability of developing PTSD.

## Key Points

• Susceptibility to developing a PTSD-like phenotype exists in animals. It can be identified behaviorally and therefore can be studied.
• Susceptibility can be studied by identifying susceptibility factors (pre-trauma) and sequalae factors (peri- and post-trauma) to a PTSD-like phenotype.
• Some susceptible individuals have impaired functioning in the hippocampus BEFORE emotional trauma.
• Understanding susceptibility can inform ways to successfully build resilience.
• Investigations into susceptibility also highlight the important idea that susceptibility is a dynamic feature of PTSD. Susceptibility is not strictly determined by genetics but requires an interaction with environmental factors, making it modifiable over time.

## Introduction

Post-Traumatic Stress Disorder (PTSD) is a debilitating condition that develops in a subset of people who experience a traumatic event, with or without physical trauma. People with PTSD display a set of symptoms often including flashbacks, invasive thoughts, nightmares that cause disruptions in activities of daily living and personal relationships (American Psychiatric Association [APA], 2013). While it is normal and adaptive to have an acute stress response after a trauma, symptoms with high severity which persist longer than 1 month result in a diagnosis of PTSD (American Psychiatric Association [APA], 2013). PTSD has been called by many names over the years from Irritable Heart during the United States Civil War to Shell Shock and Combat Stress during the World Wars. The current name, PTSD, was coined during the Vietnam War and was officially recognized in 1980 in the *DSM III*. Although about half of all people will experience trauma in their lives, only some exposed individuals will develop PTSD, suggesting that there are factors leading to susceptibility (Dursa et al., 2014). Notably, PTSD is also a sexually dimorphic condition with women being twice as likely to develop PTSD as men: lifetime prevalence is 5% for men and 10% for women (Kessler et al., 1995; Haskell et al., 2010).

Developing animal models to study PTSD is essential because they can provide insights about its pathogenesis as well as help develop new treatments. Searching for new treatments is important because existing treatments are either effective but have a high attrition rate (Resick et al., 2002) or have better compliance but moderate to low efficacy (Cusack et al., 2016). In addition, people exhibiting distinct clusters of symptoms respond differently to different treatment types (Korte et al., 2016). While these considerations underscore the importance of finding new effective treatments, they also highlight the importance of preventing PTSD from developing in the first place. Revealing susceptibility factors that can be manipulated before trauma can help develop strategies to build resilience and decrease the probability of developing PTSD. The focus of this review will be on animal studies that model psychogenic trauma and investigate the susceptibility state, and its underlying factors, before, during or after trauma.

## Definition of Susceptibility

**FIGURE 1:**
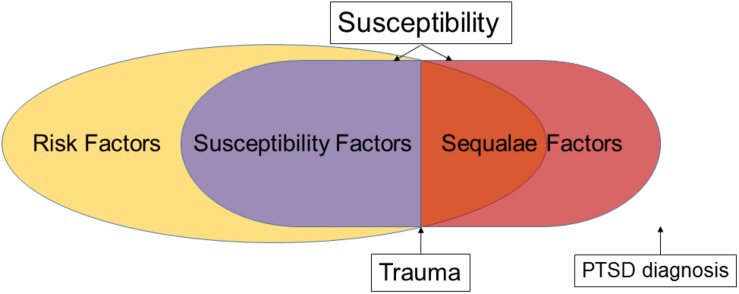
Theoretical framework of the relationship between risk, susceptibility, and sequalae factors. Susceptibility is a state that can be altered by manipulating susceptibility and/or sequalae factors.

The term “susceptibility” to developing PTSD has been used in various contexts, including in various animal models. As discussed below, susceptibility has been investigated either by identifying risk factors, or by studying individual differences in behavior and stress responses before, during, or shortly after trauma. We believe that providing an operational definition of susceptibility and underlying factors will facilitate scientific inquiry. We also emphasize that an ontological distinction should be made between factors identified before or after trauma, because this knowledge can guide the development of time-appropriate interventions. Therefore, we propose that susceptibility is a state that is influenced by a subset of risk factors, termed susceptibility, and sequalae factors (Figure 1). Risk factors comprise a broad category that includes biological or social factors, such as sex and economic disadvantage that can predispose an individual to PTSD but, given a trauma, do not necessarily result in the development of the disorder, nor are they easily manipulated. Susceptibility factors, on the other hand, are a subset of risk factors with the following key features: (1) they are detectable before trauma, (2) they can be manipulated, and (3) manipulation of susceptibility factors alters the likelihood of developing PTSD, thus affecting resilience. Manipulation of susceptibility factors can affect how an individual perceives a traumatic experience. This suggests that effectively addressing susceptibility factors before trauma will result in proper response to and encoding of the traumatic event. This, in turn, can decrease the likelihood of developing PTSD. The above definition of susceptibility factors requires that there is a distinction between pre-trauma, and peri- or early post-trauma factors that affect the development of PTSD. We term these factors sequalae factors which have the following key features: (1) they are detectable during or shortly after trauma (but before PTSD diagnosis), (2) they can be manipulated, and (3) they drive post-trauma processes such that manipulating them during or shortly after trauma decreases the probability of developing PTSD. Thus, susceptibility is a state that is comprised of both susceptibility and sequalae factors (Figure 1). A key distinction between susceptibility factors and sequalae factors is that although both can exist before trauma, the latter only plays a role post-trauma. Therefore, manipulation of sequalae factors will be most impactful peri- or post-trauma. These distinctions are important because they will inform the development of successful targeted strategies to increase resilience and early post-trauma interventions that will minimize the development of PTSD.

## Modeling a PTSD-Like Phenotype in Animals

To study susceptibility empirically, research focuses on using preclinical rodent models to assess a PTSD-like phenotype (Milad et al., 2006; Yehuda and LeDoux, 2007; Goswami et al., 2012). Many animal models of PTSD with face and construct validity have been developed (Hartmann et al., 2012; Toth et al., 2016; Deslauriers et al., 2019). The purpose of this review is not to review these, but to identify those models that are suitable for investigating individual differences in susceptibility. Nonetheless, several points should be emphasized. (1) Many models with face validity use psychogenic stress with hippocampal involvement in the stress response (Jankord and Herman, 2008; Matar et al., 2013) but without physical trauma. Examples include: inescapable electrical shock (Nalloor et al., 2011; Bali and Jaggi, 2015), predator-stress (Zoladz et al., 2015), single prolonged stress (Liberzon et al., 1997), social defeat stress (Hammels et al., 2015), or a combination of these as in the SEFL (Stress Enhanced Fear Learning) model (Rajbhandari et al., 2018) and unpredictable variable stress model (van Riel et al., 2003). Rodents subjected to psychogenic trauma exhibit one or more PTSD-like symptoms identified in the *DSM 5* symptom clusters described in Tables 1, 2) The behavioral correlates to PTSD symptom clusters can be reliably evaluated in animals via well-established tests of anxiety-like behavior, hyperarousal, cognitive function, and memory (see Table 1). (3) Models with construct validity (e.g., knockout of FKBP5, COMT, or CRH overexpression in early life) do not produce a complex PTSD-like phenotype (Hartmann et al., 2012; Toth et al., 2016; Deslauriers et al., 2019). (4) Animal models can inform about some, but not all, aspects of the PTSD phenomenon. PTSD is an incredibly complex disorder with a myriad of trauma types and presentations in humans (Galatzer-Levy and Bryant, 2013). Rodent models cannot describe the more complex symptoms such as dissociation, deficits in complex social interactions, and flashbacks (American Psychiatric Association [APA], 2013; Shalev et al., 2019). In addition, rodent models cannot describe the subjective human experience of PTSD, so we rely on human descriptions of their trauma, how cultural factors motivate their behaviors, and how effectively they manage their symptoms. (5) Despite these limitations, rodent models are widely used to address basic questions about a PTSD-like phenotype such as the underlying neurobiological systems of specific behaviors and genetic/functional abnormalities that are difficult or unethical to study in humans. Therefore, carefully crafted animal models can be used to study susceptibility, as outlined in see sections “Sequalae Factors: Animal Models of Susceptibility to a PTSD-Like Phenotype Using Post- and Peri- trauma Assessment” and “Susceptibility Factors- Pre-trauma Assessment of Susceptibility With the RISP Rat Model (Revealing Individual Susceptibility to a PTSD-Like Phenotype)” below.

**TABLE 1:** *DSM 5* PTSD symptom cluster and some rodent behavior correlates.

***DSM 5* PTSD symptom cluster**	**Rodent behavior correlate**	**Relevant citations**
Intrusions (Cluster B)	Spontaneous recovery of freezing during fear extinction	Rescorla, 2004 (Review) Nalloor et al., 2011 (Rat) VanElzakker et al., 2014 (Review)
Avoidance (Cluster C)	Avoidance of trauma related cues	Vazdarjanova and McGaugh, 1998 (Rat) Philbert et al., 2011 (Mouse) Chen et al., 2012 (Rat) Villain et al., 2018 (Mouse)
Altered cognition and mood (Cluster D)	Impaired fear extinction and anxiety-like behavior (D4), impaired performance on spatial water maze, depressive-like, and anhedonia-like behavior (D5, 7)	D4 Walf and Frye, 2007 (Review) Nalloor et al., 2011 (Rat) Paredes and Morilak, 2019 (Review) D5, 7 Cohen et al., 2003 (Rat) Hales et al., 2014 (review)
Altered arousal and reactivity (Cluster E)	Increased risk assessment and avoidance of open spaces (E3), elevated acoustic startle response (E4), impaired attention in set shifting (E5), disturbed sleep (E6)	E3 Goswami et al., 2012 (Rat) E4 Koch and Schnitzler, 1997 (Review) Rasmussen et al., 2008 (Rat) Nalloor et al., 2011 (Rat) Matar et al., 2013 (Rat) E5 George et al., 2015 (Rat) Heisler et al., 2015 (Mouse) E6 Philbert et al., 2011 (Mouse)

*These are sample studies that are not meant to be completely inclusive.*

**TABLE 2:** Summary of classification criteria, Post-trauma phenotype and test parameters for the RISP model.

**Model**	**Pre-trauma behavior**		**Trauma**	**Post-trauma phenotype**	
	**Acoustic Startle Response (ASR1)**	**Elevated Plus Maze (EPM)**	**Contextual Fear Conditioning (CFC) with foot shock**	**Fear Extinction Training (EXT)**	**Acoustic Startle Response (ASR2)**
Resilient (RES) phenotype	*Low ASR* defined as Average ASR less than group average and six or more individual readings below group average	*Low anxiety-like behavior* measured as ≥3 entries into the open arms	Acute stress response (freezing) similar to SUS rats	RES rats exhibit *unimpaired fear extinction* (progressively lower freezing)	ASR 2 response that is the same or lower than ASR 1
Susceptible (SUS) phenotype	*High ASR* defined as Average ASR greater than group average and six or more individual readings above group average	*Heightened anxiety-like behavior* measured as ≤2 entries into the open arms	Acute stress response (freezing) similar to RES rats	SUS rats exhibit *impaired fear extinction* (slower extinction or days with re-bound freezing)	ASR 2 response that is the same or higher than ASR 1
Test parameters	3 min habituation followed by 15 trials with random inter-trial intervals (15–45 s) of 120 db white noise bursts	5 min exploration of EPM (77 cm above floor) under low light (0–6 lux) and low open arm ledge (1 cm)	3 min habituation to shock context followed by 2 × 1 mA shocks 30 s apart. 5 min total duration in box	5 days of 5 min exposure to shock context without any shock.	3 min Habituation followed by 15 trials with random inter-trial intervals (15–45 s) of 120 db white noise bursts

## Dissociating Between Risk and Susceptibility/Sequalae Factors in Humans Is Difficult

A great deal of effort has been expended to examine the genetic and physiological basis of susceptibility to PTSD. Consequently, a number of risk factors have been identified. For instance, hypothalamic-pituitary-adrenal (HPA) axis dysfunction exists in people with PTSD and has been well studied (Yehuda et al., 1995; Lindauer et al., 2006; Yehuda, 2009), yet further research has revealed that it is only a risk factor and is not predictive of developing PTSD (Cohen et al., 2006b; Lindauer et al., 2006; Morris et al., 2016). In line with HPA dysfunction, glucocorticoid receptor polymorphisms have been thoroughly investigated (Yehuda, 2009). However, the overall prevalence of these polymorphs in people with PTSD has been found to be within normal range of the population (Girgenti et al., 2017), suggesting that it is a risk factor rather than a susceptibility/sequala factor: it is a measure that is not sensitive enough to separate those who will develop PTSD from those who won’t. Another identified risk factor in humans is smaller hippocampal volume. Using MRI in twin pairs, prospective studies show that smaller hippocampal volume is positively correlated with developing PTSD (Gilbertson et al., 2002, 2006; Childress et al., 2013; Logue et al., 2018) but it is not easily manipulated and therefore is not a susceptibility factor.

Chaperone proteins, such as heat shock protein (HSP70 and HSP90), have also been shown to influence PTSD (Zhang et al., 2010), but have not been demonstrated to be a susceptibility or sequala factor. Others have examined autonomic responses such as blood pressure and heart rate variability and confirm that aberrant heart rate is a risk factor (Shalev et al., 1998; Morris et al., 2016). The 5-HTTLPR genotype was examined and it was determined that the SS genotype is associated with decreased symptom severity in people exposed to emotional abuse (Walsh et al., 2014), but it remains unclear if the LL genotype is a susceptibility/sequala factor. Early life stress and epigenetic modulation of the stress response is also noted to play a role in PTSD as risk factors (Fish et al., 2004; Uddin et al., 2010; Radley et al., 2011), but is unclear if they are susceptibility or sequalae factors. Recent Genome Wide Association Studies have shown strong genetic overlap of PTSD with schizophrenia and depression (Duncan et al., 2018; Gelernter et al., 2019) as well as strong candidate genes (Xie et al., 2013), but these have yet to be examined for their role in susceptibility. The International Consortium to Predict PTSD (ICPP) found that a combination of risk factors, e.g., female, a history of interpersonal trauma, and a high Clinician Administered PTSD Scale (CAPS) score shortly after experiencing trauma, predict a high likelihood of developing PTSD (Shalev et al., 2019). While useful for clinical decisions about early interventions, this predictability includes a measure taken after trauma, and thus susceptible individuals cannot be identified before trauma which would allow them an opportunity to build resilience.

Pre-trauma elevation in pro-inflammatory cytokines has been shown to correlate positively with the development of PTSD after combat exposure in soldiers (Lindqvist et al., 2014) and hence investigated as a putative susceptibility factor. If a pro-inflammatory state turns out to be predictive of developing PTSD after trauma, it can potentially be manipulated with pharmacological or lifestyle interventions to reduce susceptibility to developing PTSD.

## Sequalae Factors: Animal Models of Susceptibility to a PTSD-Like Phenotype Using Post- and Peri- Trauma Assessment

Among the many animal models of a PTSD-like phenotype, few have explicitly attempted studying susceptibility (Cohen et al., 2003, 2006b, 2012; van Riel et al., 2003; Adamec et al., 2006; Gruene et al., 2015; Toth et al., 2016; Rajbhandari et al., 2018; Deslauriers et al., 2019 Reviews Yehuda and Antelman, 1993; Deslauriers et al., 2018; Fenster et al., 2018). Additionally, most of the models that have assessed susceptibility have done so using varied peri- or post-trauma behaviors. A prominent model defined susceptibility and resilience as “maladapted” and “well adapted” based on behavioral responses 7 days after an intense predator exposure (Cohen et al., 2003). Cutoff behavioral criteria on the elevated plus maze (EPM) (red light, 1 cm ledge) were used such that animals with no entries into the open arms and 5 min in the closed arms were classified as maladapted and those with eight or more entries into the open arms with 1 min or less in the closed arms were classified as well adapted. This strategy models a component of PTSD where, given the same trauma, some people will go on to develop PTSD and others will not. This model allows researchers to address post-trauma sequalae and how different classifications of rats differ on various physiological measures such as corticosterone levels and heart rate. Another model combines SEFL with behavioral phenotyping to classify mice as susceptible or resilient based on peri-trauma behavior (Sillivan et al., 2017). This model shows strong impairments in extinction of susceptible mice after SEFL in addition to elevated serum corticosterone in response to a dexamethasone suppression test, indicating prolonged HPA axis dysfunction. In these and similar models, identifying and studying susceptibility occurs either during or after the emotional trauma, making these models suitable for investigating sequalae factors. Building resilience, however, may be more successful if susceptibility is identified and mitigated prior to trauma. The RISP model described below was developed in an effort to identify susceptibility prior to trauma and therefore allow researchers to study susceptibility factors that can be targeted to build resilience.

## Susceptibility Factors- Pre-Trauma Assessment of Susceptibility With the Risp Rat Model (Revealing Individual Susceptibility to a PTSD-Like Phenotype)

**FIGURE 2:**
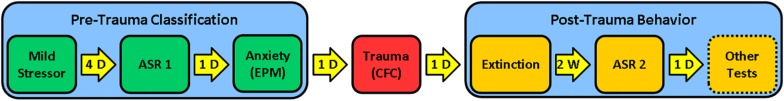
RISP model: *Stage 1 (Pre-trauma classification)*: Rats are briefly stressed with a mild stressor (a ball of cat hair). Four days later, they are classified as Resilient (RES), Susceptible (SUS), or Intermediate (Int) based on their acoustic startle response (ASR1) and anxiety-like behavior in the Elevated Plus Maze (EPM); *Stage 2 (Psychogenic trauma)*: Rats experience foot shock-motivated contextual fear conditioning (CFC) as a psychogenic trauma; *Stage 3 (Post-trauma phenotype)*: Fear extinction and post-trauma startle (ASR2) are assessed. The original presentation of the model does not include other tests aligned with a complex PTSD-like phenotype, but these can be performed to evaluate cognitive and anxiety-like behavior after trauma. D = day; W = week.

The RISP model, which incorporates both face and predictive validity, was developed to study susceptibility factors prior to trauma (Nalloor et al., 2011). The model consists of three stages (Figure 2): (1) Pre-trauma classification (acoustic startle response and anxiety-like behavior) to identify animals as susceptible (SUS) or resilient (RES) to a PTSD-like phenotype after subsequent trauma, (2) trauma (inescapable foot shock), and (3) post-trauma PTSD-like behaviors (impaired fear extinction and lasting elevation in acoustic startle response). This model was developed with an outbred strain of male rats (Sprague-Dawley).

### Pre-trauma Classification

In the first stage of the RISP model, rats are classified as SUS, RES or Intermediate, based on startle and anxiety-like responses 4–5 days after exposure to a mild stressor (a ball of cat hair, CH). Encountering the CH for a brief time (3 min) is stressful, but not traumatic. Exposure to predator/predator odor shows a dose response effect from mild to traumatic depending on duration, intensity, and number of exposures (Adamec and Shallow, 1993; Yehuda and Antelman, 1993; Vazdarjanova et al., 2001; Adamec et al., 2006; Cohen et al., 2006a; Siegmund and Wotjak, 2006; Zoladz et al., 2008; Muñoz-Abellán et al., 2009; Mackenzie et al., 2010). Under the conditions outlined above, it is not traumatic, and we describe it as a “mild stressor” because it does not induce contextual fear conditioning (CFC) (Vazdarjanova et al., 2001; Nalloor et al., 2011). Although in the RISP model we use CH, any mild stressor that elevates corticosterone and does not induce CFC should be effective in revealing susceptibility.

The use of a mild stressor before classification is necessary to reveal susceptibility. We have shown that without CH exposure susceptibility cannot be revealed using the same criteria. Only 1 of 71 rats was classified as susceptible using the criteria (Table 2) that work well in identifying susceptible (15–25%) and resilient (25–35%) rats after exposure to a mild stressor (Nalloor et al., 2011). In addition, a mild stressor prior to trauma improves the face validity of the model. It puts an animal in a state of elevated stress which is akin to people living in high stress environments or those with high stress occupations, two groups who have higher rates of PTSD (Skogstad et al., 2013).

It is important to note that the magnitude of the fear response to the mild stressor (CH) cannot predict the recovery from a subsequent emotional trauma, but classification after the mild stressor can (Nalloor et al., 2011). A related point is that the stress response elicited by the CH does not directly affect susceptibility classification. Animals are allowed to rest for 4 days before assessing susceptibility (Nalloor et al., 2011) while corticosteroid levels return to baseline by 24 h (Roozendaal, 2000).

Susceptibility assessment is performed on the 4th and 5th days, based on the rats’ acoustic startle response (ASR 1) and anxiety-like behavior on the EPM. Rats with high ASR 1 and low EPM open arm entries are classified as susceptible (SUS) and rats with low ASR 1 and higher EPM open arm entries are classified as resilient (RES) (see Table 2 for specific details). The order of testing ASR and EPM does not affect the susceptibility assessment, however, the parameters of the EPM do. Lighting conditions, height of the ledge on the open arms, and elevation from the floor all affect the number of arm entries (Fernandes and File, 1996). EPM and ASR measures were selected and treated as independent measures because they target two different brain systems (Davis et al., 1982; Walker et al., 2003). Consistent with the notion that EPM and ASR are independent measures, 45–55% of animals put through this paradigm are classified as intermediate, meaning that they have high ASR and high EPM open arm entries or low ASR and low EPM open arm entries. These intermediate animals are excluded from analysis.

### Trauma

The second stage of the RISP model is inducing psychogenic trauma. The day after classification, rats undergo CFC (see Table 2 for parameters). Foot shock in a novel place was chosen because it induces emotional trauma without physical trauma and it allows us to control stimulus intensity and timing. To the rats, it is unexpected and inescapable and therefore engages stress response systems (Bali and Jaggi, 2015). It is the most commonly used trauma in PTSD preclinical research which allows findings from the RISP model to be compared with those obtained using other models. One procedural difference from other models is that we deliver foot shocks through solid floor plates which results in more intense fear conditioning than commonly used grid floors (Vazdarjanova and McGaugh, 1998). The acquisition of fear conditioning is assessed by measuring freezing, a rodent behavior displayed readily when male rats are in a state of intense fear. It should be stressed, that we and others have shown that peri-trauma classification based on freezing during CFC training cannot predict the post trauma sequalae (LeDoux, 1998; Nalloor et al., 2011).

### Post-trauma PTSD-Like Behavior

**FIGURE 3:**
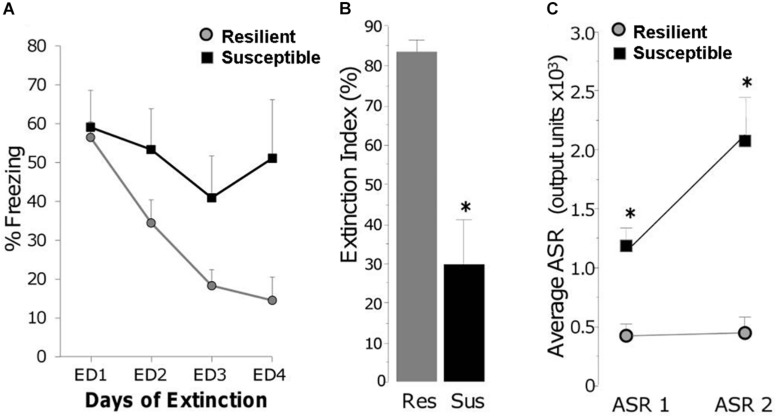
**(A)** Freezing during daily extinction sessions of Resilient (gray circles) and Susceptible (black squares) rats. **(B)** Magnitude of extinction on day 4 (ED4) in Resilient (RES) and Susceptible (SUS) rats which is the percent reduction in freezing from ED1 to ED4; ^∗^ *p* < 0.01. **(C)** Acoustic startle response at classification (ASR 1) and 3 weeks post trauma (ASR 2); ^∗^ *p* < 0.001. From “Predicting impaired extinction of traumatic memory and elevated startle,” by Nalloor et al. (2011). Copyright 2011 by the PLoS One. Adapted with permission.

The third stage of the RISP model is assessing the post-trauma PTSD-like phenotype. On the day after fear conditioning, rats begin extinction training for several days, which consists of exploration of the shock apparatus for 5 min per day without foot shock. Extinction is conducted until RES animals show less than freezing in the shock context (4–5 days, Figure 3A). SUS rats have impaired extinction compared to RES rats (Figures 3A,B), which is often seen in people with PTSD (Milad et al., 2008; Jovanovic et al., 2012) and is used as a rodent correlate of diagnostic clusters B (Intrusions) and D (Altered cognition and mood) (Table 1). Additionally, fear extinction training is recognized as the rodent correlate for Prolonged Exposure Therapy and is used in some models as such (Paredes and Morilak, 2019). We use short durations of daily fear extinction sessions to assess learning of safety across days, as measured by reduced freezing.

To investigate several aspects of the PTSD-like phenotype, the RISP model includes assessing ASR a second time (ASR 2) 1 week after completing extinction. We have reported that ASR2 in SUS rats is similar or higher compared to ASR1 (Figure 3C). Sustained elevated startle response is one of the human diagnostic criteria for PTSD (cluster E – Altered arousal and reactivity). Although the post-trauma phenotype of the RISP model includes extinction and ASR, we are currently testing whether it extends to behaviors representative of other diagnostic criteria (Table 1) such as performance on the spatial water maze or in the set shifting task.

## Impaired Hippocampal Function as a Putative Susceptibility Factor Identified With the Risp Model

### Rationale for Investigating Impaired Hippocampal Function as Putative Susceptibility Factor

Although PTSD symptoms suggest stronger and longer-lasting encoding of emotion-influenced information (Cahill and McGaugh, 1998; McGaugh, 2004; Roozendaal et al., 2006), PTSD patients often have fragmented recollections of the traumatic event which lack integrated environmental perceptions (McFarlane, 2000), even to the degree of trauma-related amnesia (Williams, 1994). This apparent discrepancy can be explained by the fact that during a behavioral experience the brain acquires different types of memories, explicit/declarative and implicit, supported by different brain systems (Packard et al., 1989; Squire et al., 2004; Manns and Eichenbaum, 2006; Ranganath, 2010).

Mounting evidence suggests that PTSD patients have general deficits in declarative and short-term memory (Bremner et al., 1993a, b, 1995; Yehuda et al., 1995; Jenkins et al., 1998; Gilbertson et al., 2001). Considering the overwhelming evidence that the hippocampal system (medial temporal lobe system) supports declarative memory (Packard et al., 1989; Squire et al., 2004; Manns and Eichenbaum, 2006; Ranganath, 2010), it is not surprising that people with PTSD have attenuated capacity for hippocampal activation during associative tasks (Rauch et al., 2006; Shin and Liberzon, 2010). It is currently unclear whether such alterations in hippocampal function are susceptibility factors that affect how traumatic memories are processed at the time of trauma by susceptible individuals, or if they are sequelae factors that affect learning of safety and expression of fear after the traumatic memory is acquired. Structurally, a smaller hippocampal size exists before trauma as revealed by studies of identical twins discordant for combat exposure and PTSD (Gilbertson et al., 2002; Childress et al., 2013). This structural abnormality may be related to functional alterations as well because cognitive impairments, including some that involve hippocampus, exist in individuals with PTSD and their non-combat exposed monozygotic twins while cognitive impairments are not seen in combat exposed non-PTSD twins and their non-combat exposed brothers (Gilbertson et al., 2006). Combined, these findings suggest that impaired hippocampal function may be a putative susceptibility factor that can be manipulated before trauma to increase resilience.

Assessing whether impaired hippocampal function is a susceptibility or sequalae factor can be accomplished with the help of animal models. Although it has been argued that declarative memories are uniquely human (Tulving, 2001), a large body of evidence exists to support the notion that essential aspects of declarative memory, specifically episodic memories, exist in rodents and depend on the hippocampal system (Eichenbaum, 2001; Morris, 2001; Babb and Crystal, 2006; Ferbinteanu et al., 2006; Kart-Teke et al., 2006; Hoge and Kesner, 2007; Li and Chao, 2008). Therefore, we have employed the RISP model to test the hypothesis that impaired hippocampal function is a susceptibility factor.

### Impaired Function of the Hippocampus Is a Putative Susceptibility Factor

To test whether a functional impairment of the hippocampus exists and can be identified before trauma, we used the RISP model and assessed expression of plasticity-related immediate-early genes, *Arc* and *Homer1a*, using the *Arc/Homer1a* catFISH (cellular compartmental analysis of temporal activity using florescence *in situ* hybridization) technique (Vazdarjanova et al., 2002). Briefly, the catFISH method (Guzowski and Worley, 2001) identifies plasticity-induced neural activity from two distinct events at the level of individual neurons and neuronal ensembles using plasticity-related immediate-early genes with suitable temporal dynamics of expression, such as *Arc* and *Homer1a* (Vazdarjanova et al., 2002). When rats explore a box (event 1) transcription of *Arc* and *Homer1a* is initiated. If the rats are put back in the same box 25 min later (event 2) another round of *Arc* and *Homer1a* expression is initiated. Due to *Arc* and *Homer1a* expression dynamics and the probes used to detect them, the presence of *Homer 1a* foci of transcription in neuronal nuclei identify neuronal ensembles responding to event 1, while the presence of *Arc* foci identifies neurons responding to event 2. Findings from *Arc/Homer1a* catFISH (Guzowski et al., 1999; Vazdarjanova et al., 2002; Vazdarjanova and Guzowski, 2004; Ramírez-Amaya et al., 2005; Nalloor et al., 2012), combined with evidence from electrophysiological studies (McNaughton et al., 1989; Barnes et al., 1990; Lee et al., 2004; Leutgeb et al., 2004), have shown that exploring the same place twice engages largely overlapping hippocampal neuronal ensembles. Changes in any feature of Vazdarjanova and Guzowski (2004) or the experience in Moita et al. (2004) and Nalloor et al. (2012) that environment results in changing the composition of neuronal ensembles, a partial “remapping,” such that, during the second experience, only part of the original ensemble gets reactivated along with neurons not activated the first time. Thus, a high degree of overlap between neuronal ensembles activated during two nearly identical events (exploring the same place twice) indicates proper hippocampal function.

**FIGURE 4:**
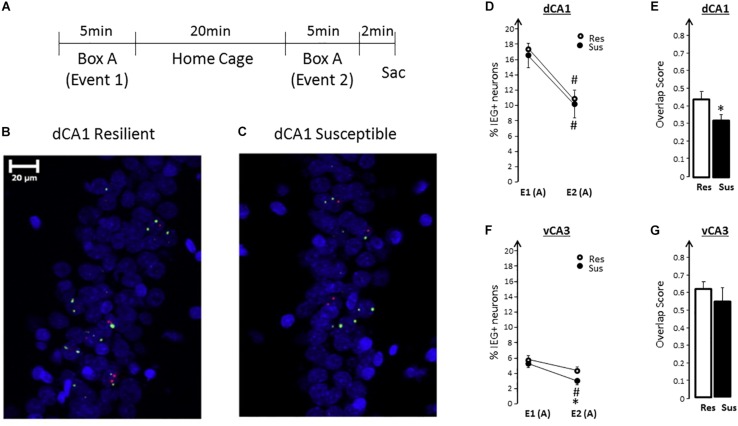
**(A)** Experimental design for spatial exploration (A-A). **(B,C)** Representative images of neuronal *Arc* (red) and *Homer1a* (green) expression in dCA1 of Resilient (RES) and Susceptible (SUS) rats using FISH. Nuclei are counterstained with DAPI (blue). **(D)** Exploring a novel context twice (box A), leads to a significant decrease in the size of IEG-expressing neuronal ensembles during the second exploration in RES and SUS rats. [Event effect *F*(1,13) = 111.68, *p* < 0.0001, no significant group effect and no significant group × event interaction]. **(E)** In SUS rats, qualitatively different dorsal/septal CA1 neuronal ensembles express plasticity-related IEGs during the second exploration (E2) compared to RES rats, as revealed by the significantly lower overlap score. [*F*(1,13) = 4.84, *p* < 0.05]. RES rats have significantly higher overlap. **(F)** There were no significant differences in the percentage of IEG-expressing ventral CA3 neurons during the initial exploration (E1): %E1 (SUS) was similar to %E1 (RES). During the second exploration, SUS rats activated smaller vCA3 neuronal ensembles: %E2 (SUS) < %E2 (RES) [*F*(1,13) = 6.37, *p* = 0.025]. **(G)** There was no significant difference in vCA3 overlap scores between RES and SUS rats. From “Predicting impaired extinction of traumatic memory and elevated startle,” by Nalloor et al. (2011). Copyright 2011 by the PLoS One. Adapted with permission. ^∗^ = SUS vs RES; ^#^ = E1 vs E2 within a group.

Rats classified as SUS or RES, without having experienced trauma, show a different degree of overlap in the hippocampus after exploring a novel place twice, 25 min apart. SUS, compared to RES, rats show less overlap of ensembles in the dorsal/septal CA1 region, as well as a smaller neuronal ensemble in the ventral CA3 region (Figure 4; Nalloor et al., 2014). These findings demonstrate that SUS rats have impaired hippocampal function (interpreted as low fidelity in episodic memory encoding) before experiencing emotional trauma. These findings identify impaired hippocampus function as a putative susceptibility factor. To classify it as a susceptibility factor, however, future studies need to show that ameliorating the functional impairment in SUS rats prior to trauma results in a post-trauma phenotype comparable to that of RES rats. Such studies are underway.

If functional impairments in the hippocampus proves to be a *bona fide* susceptibility factor, it will become essential to the prevention of PTSD to develop tests to quickly and inexpensively assess susceptibility, as well as validate interventions that will address such impairments and thus build resilience. The implications are widely applicable to populations in occupations with high-stress/high-risk for developing PTSD, as well as in situations of mass trauma exposure such as natural disasters or acts of terror/war where limited resources can be directed to have the most impact on susceptible individuals most likely to develop PTSD. Potential resilience-building approaches that improve hippocampal function include exercise, diet and “cognitive exercises” (Jeong et al., 2011; Tang et al., 2017; Firth et al., 2018).

## Future Directions

### Revealing Other Susceptibility Factors

Using the framework for susceptibility proposed in Figure 1 and see section “Definition of Susceptibility,” our lab is using the RISP model to evaluate several putative susceptibility factors, in addition to disrupted hippocampal function (as outlined above). One such factor is impaired function of the ventral medial prefrontal cortex (vmPFC, ventral-medial surface of the frontal lobe). PTSD patients have impaired behaviors supported by the vmPFC, i.e., inhibitory control over behavior, including impaired fear extinction (Milad et al., 2008; Jovanovic et al., 2012) and cognitive flexibility (Gilbertson et al., 2001; Kanagaratnam and Asbjørnsen, 2007). Given that the vmPFC is involved in context dependent emotional regulation and consolidation of fear extinction (Milad et al., 2006; Kesner and Churchwell, 2011; Euston et al., 2012; Cassaday et al., 2014), it is not surprising that people with PTSD have attenuated recruitment of this region when active suppression of trauma-related information is required (Rauch et al., 2006; Shin and Liberzon, 2010). Again, because these studies are performed after PTSD diagnosis, it is unclear if impaired function of the vmPFC is a susceptibility or sequalae factor. Investigating this distinction in rodents is possible because fear extinction, as well as the therapeutic effect of experiencing fear extinction, depend on the rodent mPFC (prelimbic and infralimbic cortex) (Milad et al., 2006; Fucich et al., 2018). Using the RISP model and *Arc/Homer1a* catFISH we are currently testing the hypothesis that impaired mPFC function is a susceptibility factor. Should the hypothesis be supported, it will suggest interventions that can build resilience. For example, improving hippocampal and mPFC function of susceptible rats by engaging them in behavioral tasks that involve both regions should increase their resilience as measured by a post-trauma behavioral phenotype, similar to that of resilient rats. We have initiated experiments to test this hypothesis by subjecting SUS rats to a series of set shifting tasks (George et al., 2015; Heisler et al., 2015) before trauma and measuring their post-trauma PTSD-like phenotype, as well as learning-induced expression of plasticity-related immediate early genes.

Another putative susceptibility factor is an increased pro-inflammatory state. Inflammation is elevated in men with PTSD and, as mentioned earlier, is positively correlated with developing PTSD in a cohort of combat exposed soldiers (Lindqvist et al., 2014). We are currently investigating whether inflammation, peripherally or centrally in hippocampus and mPFC, is a susceptibility or sequalae factor. If it is a susceptibility factor, then: (1) decreasing inflammation before classification will results in fewer rats classified as susceptible, and (2) decreasing inflammation in susceptible rats prior to trauma will build resilience to developing a PTSD-like phenotype. If it is a sequalae factor, then decreasing inflammation after trauma will reduce symptom severity.

### Developing a Female Animal Model to Study Susceptibility Factors

Post-Traumatic Stress Disorder is a sexually dimorphic condition such that women are affected with PTSD at twice the rate of men (Kessler et al., 1995; Haskell et al., 2010; Christiansen and Elklit, 2012; Charak et al., 2014; Gamwell et al., 2015; Garza and Jovanovic, 2017; Meyer et al., 2018). While female sex remains a risk factor, the sexual dimorphism present in the acquisition (Inslicht et al., 2013), presentation (Olff et al., 2007; Hourani et al., 2015), and treatment efficacy (Blain et al., 2010) of PTSD suggests that there are underlying biological differences based on sex that need to be investigated separately in both sexes (Cahill, 2003a,b, 2006; Pineles et al., 2017). Similarly to humans, rodent models of PTSD-associated behaviors show sex differences in response to trauma (Faraday, 2002; Adamec et al., 2006; Graham et al., 2009; Cohen and Yehuda, 2011; Toth et al., 2016; Pineles et al., 2017; Deslauriers et al., 2019). The RISP model was developed in male rats and we hypothesize that female rats will require different behavioral parameters for classification and PTSD-phenotyping. Female Sprague-Dawley rats tend to freeze less than their male counterparts (Wilson et al., 2013; Gruene et al., 2015). Therefore, freezing may not be the best measure to assess safety learning (extinction) in female rats. Based on previous work with female rodents we expect that avoidance will be a better measure of fear conditioning extinction (Wilson et al., 2013; Toth et al., 2016). We are currently working on modifying the RISP model with more ethologically relevant tasks for females to determine what a PTSD-like phenotype is for female rats and how we can predict it. At present, it is unknown whether there are differences in susceptibility factors based on sex. Having the appropriate tools will then allow us to determine if susceptibility is the same in males and females. If susceptibility and sequalae factors prove to be sexually dimorphic as well, that will have profound implications for building resilience strategies, as “one size fits all” interventions will be sorely inadequate.

## Final Thoughts and Implications

Revealing susceptibility to identify successful resilience-building strategies and prevent PTSD from developing in the first place is imperative, because PTSD currently has no cure and exposure to trauma is unpredictable. Susceptibility exists because only a subset of the population develops PTSD. Here we have proposed a distinction between risk, susceptibility, and sequalae factors to provide an integrated theoretical framework to help with experimental investigations (Figure 1 and see section “Definition of Susceptibility”). Risk factors exist before trauma and may increase the probability of developing PTSD, but may not always be easily manipulated. Susceptibility is a state that is influenced by susceptibility and sequalae factors. Susceptibility factors are a subset of risk factors that can be detected before trauma, can be manipulated, and amelioration will result in decreased probability of developing PTSD after trauma. Similarly, sequalae factors are those that may or may not exist before trauma but play a key role after trauma. Existing models that study PTSD preclinically have primarily addressed risk and sequalae factors. The RISP model allows us to study susceptibility and sequalae factors because it classifies rats as resilient or susceptible to a PTSD-like phenotype prior to trauma. Using the RISP model we have identified one putative susceptibility factor: impaired hippocampal function before trauma as measured by expression of the plasticity-associated genes *Arc* and *Homer1a*.

Furthermore, we propose that susceptibility is a dynamic feature of PTSD that is subject to change based on life circumstances and history of trauma. People may change their life circumstances, e.g., get a meaningful job or get divorced, which can alter stress levels and other factors that can affect the state of susceptibility. Consistent with this idea are findings that people who have jobs with high baseline levels of stress are more likely to develop PTSD after exposure to trauma (Skogstad et al., 2013). As the development of the RISP model shows, a mild stressor is necessary to reveal susceptibility (Nalloor et al., 2011) (also see section “Pre-trauma Classification”). Therefore, susceptibility is a state that can be altered. This implies that an individual’s state of susceptibility does not have to result in PTSD; rather, by targeting identified susceptibility and sequalae factors in an individual, resilience can be built before trauma or early post-trauma interventions can be given to decrease the probability of developing this crippling condition. This is especially important in light of the findings that increased number of traumatic experiences lead to increased probability of developing PTSD and comorbid diseases (Duncan et al., 1996; Sledjeski et al., 2008; Lewis et al., 2019). Thus, resilience to one trauma may not indicate resilience to subsequent traumas, so susceptibility needs to be evaluated after each trauma and mitigated, if needed.

## Author Contributions

KA and AV wrote the manuscript with input from all authors. RN, KB, and AV developed the RISP model. AV acquired funding. All authors read the final version of the manuscript.

## Conflict of Interest

The authors declare that the research was conducted in the absence of any commercial or financial relationships that could be construed as a potential conflict of interest.
